# Perspectives from primary health care providers on their roles for supporting adolescents and young adults transitioning from pediatric services

**DOI:** 10.1186/s12875-020-01189-8

**Published:** 2020-07-13

**Authors:** Kyleigh Schraeder, Gina Dimitropoulos, Kerry McBrien, Jessica Yijia Li, Susan Samuel

**Affiliations:** 1 Department of Pediatrics, Cumming School of Medicine, University of Calgary, 28 Oki Drive, Calgary, Alberta Canada; 2 Faculty of Social Work, University of Calgary, Calgary, Alberta Canada; 3 Mathison Centre for Mental Health Research & Education, University of Calgary, Calgary, Alberta Canada; 4 Department of Family Medicine, Cumming School of Medicine, University of Calgary, Calgary, Alberta Canada

**Keywords:** Adolescent, Young adult, Transition to adult care, Primary care, Family physicians, Patient-centered care, Delivery of health care, Chronic disease, Qualitative methods

## Abstract

**Background:**

Transitioning from pediatric care to adult-oriented care at age 18 (the age of transfer in most countries and jurisdictions) is a complex process for adolescents and young adults affected by chronic physical health and/or mental health conditions. The role of primary health care (PHC) providers for this population is poorly understood. Perspectives from these providers, such as family physicians and other members of the primary care team, have not been explored in depth.

**Methods:**

A total of 18 participants (e.g., family physicians, social workers, nurses) were recruited from 6 Primary Care Networks in Calgary, Alberta, Canada. Semi-structured individual interviews were conducted, and transcribed verbatim. A qualitative description approach was used to analyze the data, and included thematic analysis.

**Results:**

Five distinct, yet overlapping, roles of primary health care providers for adolescents and young adults transitioning to adult care resulted from our analysis: (1) being the “*common thread*” (continuous accessible care); (2) caring for the “*whole patient*” (comprehensive care); (3) “*knowing families*” (family-partnered care); (4) “*empowering*” adolescents and young adults to develop *“personal responsibility”* (developmentally-appropriate care); and (5) “*quarterbacking*” care (coordination of specialist and/or community-based care). Participants identified potential benefits of these roles for adolescents and young adults transitioning to adult care, and barriers in practice (e.g., lack of time, having minimal involvement in pediatric care).

**Conclusions:**

Input from family physicians, who follow their patients across the lifespan and provide the majority of primary care in Canada, are critical for informing and refining recommended transition practices. Our findings provide insights, from PHC providers themselves, to bolster the rationale for primary care involvement during transitions from pediatric specialty and community-based care for AYAs. Solutions to overcome barriers for integrating primary care and specialty care for adolescents and young adults need to be identified, and tested, with input from key stakeholders.

## Background

For many adolescents and young adults (AYAs) living with a chronic health condition (e.g., diabetes, cystic fibrosis) and/or long-lasting mental illness (e.g., depression, eating disorder), the transition from a pediatric- to adult-oriented care system is a complex and challenging process. As AYAs approach the age of transfer (typically age 18, though this varies across jurisdictions), their regular care provider shifts from a pediatric specialist (and/or general pediatrician) to a family physician; adult specialists may also become involved. These transfers or ‘hand-offs’ between providers can lead to disruptions in care for AYAs [1, 2]. For example, AYAs transitioning to new adult providers may experience delays in treatment, deterioration in their health and/or mental health, and disengagement from healthcare services [3, 4]. The importance of continuous primary care (by a family physician) has been recommended in best practice transition guidelines [5–7]. Family physicians, who follow their patients and families across the lifespan, may be uniquely equipped to provide transition care for AYAs, but this has not been adequately studied [8–10]. Understanding the roles and involvement of family physicians, and other members of the primary care team, for AYAs with chronic conditions is criticial for developing and informing effective models of transition care.

There are a number of reasons why having a family physician may be beneficial for AYAs transitioning from pediatric services [11]. First, primary care is generally viewed as more accessible than specialist healthcare services, and may be convenient for AYAs to receive routine follow-up care than adult specialist clinics [12]. Family physicians also offer continuity of care to their patients, and can act as a “trusted key person” during the transition period, as described in research involving long-term pediatric cancer survivors [13, 14]. Recent work has shown AYAs with diabetes who have continuous primary care (or no gaps in primary care) during the transition age (i.e., 17 to < 19 years old) may have a lower risk of experiencing adverse outcomes in young adulthood (e.g., hospitalizations, diabetes-related admissions) [15]; similar findings have also been demonstrated for AYAs with severe mental illness [16]. Much of the focus in the transition literature has been on transfers between child- and adult-specialists or subspecialists [17–20], and not on what happens in primary care. A recent systematic review of pediatric transition interventions yielded only three studies with a primary care component [21]; none evaluated this component specifically. There is currently little empirical evidence to guide practice on the role of family physicians during the transition process.

Clarity about the roles of family physicians is needed for AYAs and caregivers. From the perspective of AYAs with chronic conditions, many are unclear about why and when primary care is needed [17, 22]. Numerous barriers to primary care for AYAs have been described, including inadequate time during appointments to address complex issues [23, 24] and a perceived lack of knowledge among PHC providers about managing specific pediatric conditions and mental health issues [25, 26]. Perspectives from family physicians themselves are needed to clarify their role(s) for supporting AYAs during the transition from pediatric to adult care. The purpose of this study was therefore to gain an understanding about the roles of family physicians, and potential barriers and facilitators to their involvement during the transition period, from the perspective of family physicians and other Primary Health Care (PHC) providers.

## Methods

### Study design and setting

We used a qualitative description design, as it focuses on describing and exploring a topic of interest, rather than generating a theory from data [27–29]. Qualitative description aims to provide a rich, detailed account of participants’ experiences and processes in the their own language, and is ideally suited when the existing literature is limited [30, 31]. This study was approved by the University of Calgary Conjoint Health Research Ethics Board (REB 17–2397). This study followed the Consolidated Criteria for Reporting Qualitative Studies (COREQ) guidelines for qualitative research (see Additional files 2).

This study was conducted in Calgary in the province of Alberta, Canada, where nearly all primary care is delivered by family physicians within Primary Care Networks (PCNs) [32–34]. PCNs enable the ‘medical home’ in Alberta by providing family physician-led clinics with access to multidisciplinary supports (e.g., dieticians, social workers, nurses). There are 42 PCNs in Alberta, with approximately 3800 physicians and 1000 other PHC professionals. Calgary has seven PCNs with 1700 family physician members, serving a catchment population of 1.4 million.

### Participants and sampling strategy

Eligible participants were: (i) PHC providers with direct experience treating or managing AYAs with chronic conditions, that (ii) could be interviewed in English. Participants were recruited through study advertising (e.g. internal posting boards, electronic newsletters) in various networks, including local academic and clinical departments of family medicine and PCNs. Purposeful sampling was used to gain perspectives from PHC providers with a range of experiences caring for AYAs with chronic conditions. Snowball sampling techniques [35] were also used to recruit additional participants with relevant experiences. Interested participants contacted the research team directly by email, or were introduced by other participants. Participants signed a consent form prior to scheduling the interview and, after the interview, received a $50 gift-card in appreciation of their time. Participants were not known to the interviewer prior to their participation.

### Data collection and analyses

Semi-structured interviews were conducted in-person or by telephone with participants between June and October 2018 by the primary author (KS), who had qualitative research training. Prior to the interview, participants were introduced to the interviewer and completed a demographics survey (e.g., age, training background). Interview questions covered participants’ experiences caring for AYAs with chronic conditions and perceived barriers and facilitators to care. Interviews were audio-recorded and transcribed verbatim; identifying information was removed from transcripts to ensure participants’ confidentiality. Data collection and analyses occurred simultaneously and iteratively. Authors (KS, JL) read and re-read all transcripts separately and together; a qualitative methodologist in our team (GD) also reviewed transcripts and provided input throughout the analytic process. We first conducted a content analysis [31], coding “straight descriptions” from the data about what participants said or believed. Two authors (KS, JL) developed an initial coding template, discussed with co-authors, and iteratively modified and refined the template, before entering it into NVivo [36]. We also followed Braun and Clarke’s [37] steps and conducted an inductive thematic analysis [37, 38], whereby meaningfully related codes were combined to create categories, subthemes, and themes. Themes represented frequently recurring content and infrequent, yet significant and novel, content (e.g., divergent perspectives) [29, 39]. Constant comparison techniques [40], comparing experiences within and across participants, were used to develop broad conceptual categories. Our research team met regularly to discuss participant recruitment and the importance of obtaining diverse perspectives to power our findings. For example, an additional physician practicing in a rural area was recruited to explore potential differences in themes across geographic locations. We discontinued data collection when it was agreed within our team that the information obtained from participants was sufficient to fulfill our study aim. Sample size was deemed adequate in our team when the data sufficiently answered our research question [41, 42].

### Validity

Rigor and validity were maximized in several ways. Our interview guide incorporated feedback from content experts (SS, GD) and family physicians (KM) in Alberta. We sampled participants in various professional roles to gain a comprehensive understanding of the topic [43]. Transcripts were checked carefully for accuracy, and three authors (KS, JL, GD) independently reviewed transcripts and collaboratively developed and refined themes. Our analysis was enhanced by triangulation techniques [40] by our interdisciplinary team, comparing interpretations between experts in primary care (KM, KS), vulnerable youth populations (GD), pediatrics (SS), and mental health (KS). Reflexivity processes, such as attending to preconceptions brought into the project and memo-writing, accounted for our influence on the findings as researchers [43]. We presented our findings to stakeholder groups as a form of ‘member-checking’, to verify the accuracy of our analysis [44]. Finally, our analytic approach involved minimal interpretation, ensuring greater fidelity to participants’ verbatim accounts.

### Final sample

A total of 18 participants were interviewed. Of the 32 individuals who expressed interest in participating, two were not eligible (i.e., not PHC providers) and 12 did not respond or could not be scheduled. Interview lengths ranged from 20 to 60 min. Sample characteristics are summarized in Tables 1 and 2. Notably, 10 participants were family physicians (FP); 8 were members of the PHC team (e.g., nurse = N, social worker = SW, mental health clinician = MH). Most (*n* = 15, 83.3%) worked in an urban practice.

**Table 1:** Characteristics of sample of primary health care professionals (*n* = 18)

Characteristics	% (n)
Sex	
Female	83.3% (*n* = 15)
Age	
< 30–39	55.6% (*n* = 10)
40–49	16.7% (*n* = 3)
> 49	27.8% (*n* = 5)
Professional role	
Family physician	55.6% (*n* = 10)
Nurse/Nurse practitioner	16.7% (*n* = 3)
Social worker	16.7% (*n* = 3)
Dietician	5.6% (*n* = 1)
Psychologist	5.6% (*n* = 1)
Primary Care Network (PCN) affiliation	
Mosaic	22.2% (*n* = 4)
South Calgary	22.2% (*n* = 4)
Calgary West Central	22.2% (*n* = 4)
Calgary Foothills	16.7% (*n* = 3)
Rural or Highland	16.7% (*n* = 3)
Years of professional experience	
< 5	22.2% (*n* = 4)
5- < 10	27.8% (*n* = 5)
> 10	50.0% (*n* = 9)
PCP’s main patient population	
Adults (> 18)	22.2% (*n* = 4)
Children/adolescents (< 18)	16.7% (*n* = 3)
Both	61.1% (*n* = 11)
Number of children, adolescents, or young adults seen with complex chronic conditions	
< 10	27.8% (*n* = 5)
10–15	16.7% (*n* = 3)
> 15	55.6% (*n* = 10)
Method of children, adolescents, or young adults entrance into care^a^	
Transferred/referred from pediatric specialist(s)	50.0% (*n* = 9)
Transferred/referred from adult specialist(s)	22.2% (*n* = 4)
Patient self-referred	27.8% (*n* = 5)
Since birth or childhood	33.3% (*n* = 6)
Transferred from other family physician	22.2% (*n* = 4)
Practice location	
Sub-urban	55.6% (*n* = 10)
Inner city	27.8% (*n* = 5)
Rural	16.7% (*n* = 3)

*PCPs* Primary Care Professionals

^a^PCPs indicated receiving AYAs from multiple referral sources, so percentage of cases for method of entrance into care sum to > 100%

**Table 2:** Unique characteristics of participant sample from interview data^a^

Unique characteristics	n (%)
Remuneration model	
Fee-for-service	8 (44.4%)
Salary based	10 (55.6%)
Leadership or management position (e.g., medical team lead)	3 (16.7%)
Expertise working with marginalized AYA populations	
Mental health	15 (83.3%)
Addictions	4 (22.2%)
Homelessness	1 (5.6%)
Worked at academic teaching clinic	
Belongs to more than one PCN, or works in multiple clinics	5 (27.8%)
Employed in a “unique role” for AYA (e.g., pediatric case manager)	4 (22.2%)
Offers after-school hours (for patient appointments, phone calls, etc.)	5 (27.8%)
Co-manages care with pediatricians	7 (38.9%)
Works in clinic with umbrella model/offers multiple services	9 (50%)

^a^Information was spontaneously provided by participants during interviews

## Results

**Fig. 1:**
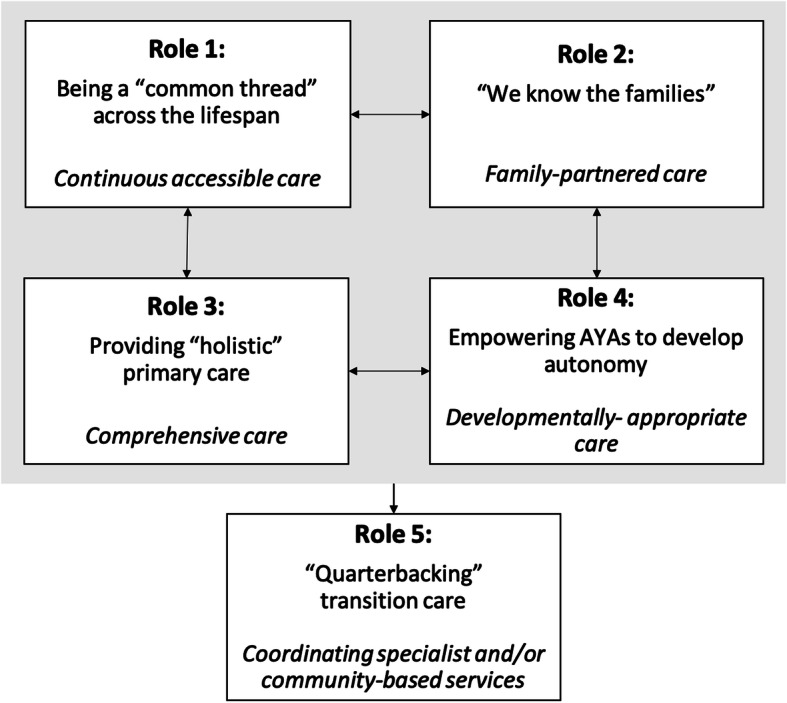
Five key roles of Primary Health Care (PHC) providers for adolescents and young adults with chronic conditions during their transition from pediatric to adult services

Our analysis yielded five distinct, yet overlapping, roles for PHC providers for supporting AYA with chronic conditions during the transition from pediatric to adult care (Fig. 1). These roles are described below with supporting quotes from the data. Perceived challenges and opportunities to improve care, organized by role, are summarized in Table 3; additional participant quotes by theme are provided in Additional file 1.

**Table 3:** Barriers and facilitators associated with Primary Health Care (PHC) roles

PHC role	Examples in practice	Barriers to this role	Facilitators to this role
Role 1: Being a “common thread” across the patient’s lifespan (*continuous accessible care*)	• Being involved as the PHC provider since birth and all life transitions • Being accessible for routine follow-up appointments (e.g., every 3–6 months) depending on condition • Managing appointments around school schedules (e.g., after-school hours, university breaks, etc.)	• AYA only followed by pediatrician prior to age 18 • Families/AYA do not attend regular appointments • Minimal involvement by family physician with specialist care • Families/AYA re-locating • Continual family physician involvement not promoted as conventional practice • AYA/family lack of understanding of role of “why” they need family physician; no “buy-in” • Lack of access to psychosocial supports in primary care	• AYA/families continue to see family physician on regular basis • Trust and long-standing relationship between AYA patient and PHC provider • Convenient appointment times for AYAs • Team-based PHC care
Role 2: Providing “holistic care” to AYA *(comprehensive team-based primary care*)	• Assessing and managing mental health issues • Discussing bullying and school • Discussing sexual health • Assessing safety (e.g., suicidal ideation) • Identifying need for supports (e.g., financial assistance, housing)	• Not knowing available resources in primary care • Age cut-offs for available psychosocial supports in primary care; e.g., Nurse only on adult side, or supports only for pediatric populations • Lack of specialist recommendations for managing within primary care • Lack of familiarity with less common, complex medical conditions (e.g., cystic fibrosis, non-verbal AYAs) • Resources not accessible for AYA (e.g., location, resource fees)	• Having multidisciplinary resources within primary care • Accessible mental health resources and supports • PHC provider role recognized/trusted by other providers on team • ‘Team’ works under one roof
Role 3: “We know the families” *(family-partnered care)*	• Caring for parents and/or extended family members of AYA • Checking-in with parents about AYA’s condition • Providing parenting supports (e.g., family counselling)	• Family members do not belong to PCN • Uncertainty with addressing legal concerns (e.g., confidentiality) • Practices not “*family friendly*” or “*welcoming*” to AYA	• Family prepared to transfer some responsibility of care to AYA patient
Role 4: “Empowering” AYA patients to develop “personal responsibility” *(developmentally-appropriate care)*	• Helping AYAs develop more responsibility for care • Teaching self-management skills • Meeting with AYAs on their own without parents • Taking a harm-reduction approach • Assessing and documenting mature minor status	• Parents who are “*challenging*” or “*will not let go*” • Lack of adolescent health specific training • Practices not “*family friendly*” or “*welcoming*” to AYA • Lack of time during appointments	• Involvement of parents/family members in process of AYA independence • Specialist providers preparing some support for the transfer to adult care
Role 5: “Quarterback-ing” for AYA *(coordinating specialist and community-based care)*	• Making referrals to specialists • Connecting patients with community-based supports • Helping patients navigate the health system • Getting a ‘team’ around patient	• Considerable time required • Lack of specialist support during coordination • No knowledge of available community resources • Lengthy wait-lists for mental health services	• Organizing clear treatment plans from other care providers • Alternate payment models to allow for time required for ‘complex’ cases

*PHC* Primary Health Care

### Role 1: Being a “common thread” across the lifespan (continuous accessible care)

Participating family physicians described themselves as a “*consistent”* provider for AYAs during childhood, adolescence, and adulthood, and *“across the lifespan”* in primary care: “*we have that ongoing relationship with [patients]”* (FP3). Participants recognized this role as distinct from other healthcare providers: *“with specialists, once you’re better you get discharged. Somebody still has to manage [AYA] long-term”* (FP16). By caring for AYAs long-term, some participants reflected the absence of any *“real transition”* in primary care: “*From a family doctor’s point of view, there is no arbitrary point where my [patients] don’t become my patients*” (FP11).

Participants believed continuous primary care was beneficial for AYAs, specifically to *“bridge the gap”* at transition: *“there’s almost no real gap in care. If, in-between [pediatric-adult care], there were any delays, [AYAs] have me who knows their treatment history … The key was being involved from the beginning*” (FP11). Participants also believed AYAs transferring to new specialists may have *“one less [new] provider”* during their transition, and a provider who *“knows”* them, who can help them work through the *“losses”* [of child-oriented providers]: *“[we] know them, will take good care of them … [we’re] not just some stranger who doesn’t understand what their life is about”* (FP16). Continuous primary care was also perceived as especially important for AYAs with mental health issues, who may encounter significant barriers to accessing adult services: *“Many kids in the adolescent mental health system do not end up in the adult system, seeing an adult psychiatrist. They stay with the family doctor because adult access to mental health services is very poor”* (FP19).

Being involved *“from the beginning”* was perceived by participants as a major facilitator to continuous primary care for AYAs. If participants were not involved prior to AYAs’ transfer, many expressed feeling *“dumped on”* by specialists; as articulated by this family physician:

*But now [at age 18], the family doctor is the primary care provider all of a sudden? For some [AYAs], I haven’t seen those kids since they were babies! It just seems like, when the kid’s 18, it’s like, “Ok, pediatrician’s done. Better go back to your family doctor.” If these kids don’t have a relationship with us, that’s a bit awkward … that’s 18 years of not seeing the kid! [laughs]* (FP7)

Participants identified potential consequences of pediatricians serving as the *“primary care provider”* for AYAs prior to age 18; for example, AYAs may assume “*the pediatrician [or sub-specialist] is there for everything”*, and thus not recognize the need to maintain relationships in primary care.

Timely access to primary care (e.g., offering evening appointments, *“working around school schedules”*), were perceived to facilitate continuous care. Participants viewed themselves as *“more accessible for follow-up”* compared to AYAs’ specialist providers: “*If [AYA] needs something [refills, prescriptions, forms], and they can’t get into their specialist, or [it’s] something not related to their specialist, then they have to come to see us”* (FP7). A social worker participant described being “*the go-to person for families”*, and *“an easy call, easier than [AYA] calling the pediatrician”* (SW18). Yet, many participants expressed frustration with*“notoriously high no-show rates”* with AYAs and difficulties *“getting them in the door”* (MH2). Thus, although participants perceived themselves to be a consistent provider, they also acknowledged potential barriers to continuous AYA engagement in primary care (e.g., clinics not *“adolescent friendly”*; see Table 3).

### Role 2: “We know the families” (family-partnered care)

Most participants described caring for AYAs’ parents and families, and viewed this as a unique “*advantage”* of their role as a PHC provider. For example, participants explained their involvement with parents as beneficial to avoiding gaps in care and staying ‘up-to-date’ with AYAs’ care: *“when parents come in and say, ‘Did you know my son has an echo and has seen a cardiologist, and we’re planning for next cardiac surgery?’ I’ll take some time at end of day and update [EMR]”* (FP7). Another participant explained asking AYAs: *“since we can’t get together in a month, can I call your mom and ask her how you’re doing in regards to your mood?”* (FP11). Caring for families was perceived as particularly beneficial for comprehensive care, as this gave participants the “*bigger picture”* or *“broader lens”* on AYA’s needs: “*Knowing the family, and caring for the family, helps you make the right decisions regarding the adolescent’s health”* (FP19). Participants also believed caring for AYAs’ families helped them gain trust among parents, and credibility among AYAs: *“I think [AYA] implicitly understands, oh okay you get what’s going on with [my family]. It’s not like talking in a vacuum”* (FP16).

Participants described various degrees of involvement with families. For some PHC team members, working with families was a primary role: *“A lot of my work is not per say with [AYAs]. My work is mostly with the parent [s] actually”* (SW18). Some family physicians described working more with parents because their AYA patients were less likely to attend primary care appointments: *“the parents are honestly more likely the ones to come in”* (FP3). All participants expressed feeling comfortable working with families, though some expressed a desire for additional training with unique aspects of AYA care (e.g., assessing mature minor status, confidentiality issues). Participants acknowledged potential dilemmas (e.g., calling Child Family Services, parent-child conflict), but these were generally accepted as *“part of the job”* in family practice: *“that’s just a challenge that exists in family medicine, any time, any age. I don’t feel it’s a downside”* (FP19).

Overall, participants recognized their role to support families of AYAs, and the benefit of having parents involved for AYAs’ own treatment adherence: *“A great deal depends on the family, and on the parents. If you don’t have buy-in from them, then how can you expect the [AYA] to buy into any of it? They can’t!”* (N10). Most felt AYAs appreciated having parents involved in their chronic care: *“often kids with chronic complex needs are quite happy for their families to be aware of what’s going on and kept informed”* (FP21).

### Role 3: Providing “holistic care” to AYAs (comprehensive primary care)

“*I think our role is to care for the whole patient”* (FP3). Participants described how their knowledge of the *“whole patient”* informed their understanding of AYAs’ clinical needs, barriers to treatment adherence, and preparedness for transition. Opportunities in primary care to monitor and assess important aspects of AYAs’ well-being were described, including: determinants of health (e.g., financial issues, housing supports), mental health and psychosocial concerns (e.g., peer relationships, learning issues, family stressors, coping skills), and *“sensitive issues”* (e.g., sexual health, contraceptive needs): *“[AYAs] don’t come to the family doctor and just get asked about their chronic condition … even if that’s the only reason they come to see you”* (FP13). This quote captured participants’ role in providing comprehensive care to AYAs, or care from a “*global, and not just medical, standpoint”* (FP5).

Some participanting PHC team members described working *“collaboratively”* or *“hand-in-hand”* with family physicians to provide comprehensive primary care. For example, one social worker participant described gathering information from the family to inform treatment planning: “*I’m kind of the puzzle keeper. I’m able to put those pieces of the puzzle together and give the [family] doctor a better history to make those decisions, to help them understand [AYA]*” (SW14). Barriers to communication in PHC teams, between family physicians and other providers, were noted to interfere with providing comprehensive care to AYAs (e.g., physical proximity between PHC providers, lack of awareness of available PHC resources).

### Role 4: “Empowering” AYA patients to develop “personal responsibility” (developmentally-appropriate, patient-centered care)

Participants described roles for “*encouraging*”, *“empowering”,* and *“enabling”* AYAs to take on more responsibility for their condition(s) as they became older. Participants recognized the transition to adult care as unique, given AYAs’ overlapping developmental transition: *“These patients are not just moving from doctor A-to-B, like a lot of adult [patients] would be”* (FP13). As articulated by one family physician, an important part of their role was therefore to empower AYAs to develop self-management skills, and focus on aspects of their care important to *them*:

“*What’s most important to the cardiologist is the heart stuff, and what’s most important to the respirologist is the lung stuff. But, I think in family medicine, I can say to [my] patient, Ok, there’s a lot of stuff going on, what’s most important to you? That’s what we can focus on.”* (FP3)

The process for AYAs to develop “*autonomy around their healthcare”*, described to start anywhere from 9 to 16 years old, was emphasized as a gradual process: *“We encourage [AYAs] to gradually take more control … its part of the whole maturation process [of] becoming adults”* (FP5). For younger AYAs, participants described *“hand-holding”, “taking the lead”* and *“not expecting [younger AYAs] to take initiative. [Because] that’s not fair to a 13-year old. If you can see they need help, you just help [and] follow-up … not let them dwindle away”* (FP11). Participants also described encouraging parents to discuss their involvement with their child’s care as they became older: *“I always encourage [parents] to chat with their child at home, and ask them what they would like. [For example] do they want a parent to come in, or would they prefer to come in on their own?”* (N10).

For older AYAs, participants explained transitioning AYAs into a *“confidential sphere”* and *“training [AYAs] to be patients”* or helping them to attend visits independently. They described gradually allowing AYAs to *“lead”* their appointments in primary care as they approach the age of transfer: *“Even if parent is present, I encourage them to be the one to contribute the most”* (FP16). Participants viewed meeting with older AYAs on their own as beneficial: *“they are allowed to have a confidential relationship with me that doesn’t involve their parent … it lets them bring up any concerns at all. They don’t have to censor themselves - I think it promotes full disclosure”* (FP5). One family physician participant held “*graduation”* visits with AYAs where they signed the family practice’s appointment policy (e.g., for cancellations/no-shows).

Participants’ role caring for families, and *“gaining parents’ trust”*, also facilitated their role of empowering AYAs: *“Parents are usually concerned about [AYAs’] transition [to] caring for their own health independently. [Parents] can trust their kid is in good hands if they know the doctor … I think that’s a pretty unique role for family doctors”* (FP19). Others described working one-on-one with parents to help them “*let go”* or *“try to get them to take that little step back … allowing their child to start taking on more responsibility”* (SW18). Importantly, participants described balancing parental involvement and AYA independence at transition.

### Role 5: “Quarterbacking” transition care (coordinating specialist and community-based services)

All participants described providing some level of care coordination at transition for AYAs with chronic conditions. Different metaphors (“*hub”, “linker”*, *“connector”,* etc.) were used to describe the wide range of coordination roles, including: identifying *“what’s available and appropriate”* (outside primary care) for AYAs, making referrals to specialist and/or community-based services, providing system-level *“navigation support”*, etc. Some participants referred to their role as the *“quarterback”* of care at transition: “*[We] make sure everybody’s on the field. If somebody’s gonna go off the field, who’s gonna replace that person if needed. That’s an integral part of our job”* (FP11).

Notably, all participants described care coordination as a “*time-consuming”* role: *“it’s a ton of time, energy … after-hours time*” (FP3). Some felt pressured to “*pick up the ball*” when AYAs turned 18 years old, with little specialist support: “*it’s like ‘family doctor, go figure it out’”* (FP11). This was perceived to be problematic when AYAs were transitioning from community-based services, for example for mental health issues: *“There’s no ‘hand-off’ … If they leave a [community] organization, they kind of drop-off. Then come back to me when they’re in their early 20s, their mental health is really poorly controlled and they’ve got active addictions”* (NP15). Other barriers to care coordination were also described, which may extend beyond the transition period (e.g., lack of remuneration, time required, lack of recommendations from specialists or community providers).

There were differing opinions in the data about how much transition care coordination should occur within primary care (versus by other providers). Some participants believed all care coordination should occur in primary care: *“I truly think family doctors are the case managers … all [patient] information should always come back to the PCN, the family doctor”* (SW18). Yet, other family physicians asserted certain aspects of care coordination should not be their role, especially if they are not involved prior to transfer; for example, with respect to referring to adult specialists: “*it should be the [provider] who is seeing them most [before age 18], so they know what [AYA] needs*” (FP2). Others believed AYA’s pediatrician should be the *“main care coordinator”* during transition*,* and then follow AYAs post-transfer. Few participants described experiences with *“shared care”*, or co-managing care with AYAs’ other providers prior to transfer. Some felt uncertain if specialists would be receptive to this model: *“I always put my cell phone number on the bottom of referrals … but I don’t know how open [specialist] physicians are to that”* (FP3). Overall, instances of collaboration and coordination during transition between PHC providers and those outside of primary care were variable among participants.

### Summary and relationship between themes

During the transition from pediatric to adult care, the five key roles identified by participants for supporting AYAs appeared interrelated (see Fig. 1). Prior to transfer, the longitudinal patient-provider relationship PHC providers develop with AYAs (Role 1) and their families (Role 2) allows them to assume Roles 3 and 4, which include providing *“holistic”* care and assessing AYAs’ transition preparedness, as well as empowering AYAs to develop autonomy and personal responsibility for their care. Participants perceived Roles 1–4 were key to facilitate their role in “*quarterbacking care*”, such as coordinating specialist and/or community-based services during the transition period (Role 5). This ‘quarterback’ role was perceived as difficult for PHC providers if AYAs *“don’t have a relationship with us”* or were not known to PHC providers prior to AYAs exiting pediatric services.

## Discussion

This study described the roles of PHC providers, such as family physicians, for supporting AYAs with chronic conditions transitioning from child-oriented care from the perspective of PHC providers. Our analysis identified five clear roles of family physicians and PHC providers during the transition period, which align with principles of care well-known to primary care (‘the medical home’) [11] and pediatric transition guidelines [6, 45]. These roles included: (1) being the “*common thread*” (continuous accessible care); (2) caring for the “*whole patient*” (comprehensive care); (3) “*knowing families*” (family-partnered care); (4) “*empowering*” AYAs to develop *“personal responsibility”* (developmentally-appropriate care); and (5) “*quarterbacking*” care (coordination of specialist and/or community-based care). Challenges with care coordination, especially between PHC providers and specialty and/or community-based services, were noted to interfere with optimal primary care during the transition period. Below, we discuss how the PHC perspective fits with existing literature.

One of the main benefits of primary care for AYAs and their families, as noted by our participants, is the opportunity for long-term patient-provider relationships. It is well documented in the literature that AYAs and families encounter barriers at transition (e.g., *“falling off a cliff”* at 18 years old, losing child-oriented providers and systems well-known to them) [46–48]. In primary care, family physicians who have cared for AYAs and their families since childhood, have the opportunity to provide families with a *“common thread”* or a needed constant during this vulnerable period of change. Family physicians, and other members of the PHC team, are ideally positioned to monitor AYA’s general health and well-being (e.g., psychosocial concerns, family stressors, coping skills) and possibly assess for transition preparedness given their knowledge of the *“whole patient”* [8]. This type of monitoring in PHC has been suggested for AYAs with chronic conditions [15, 16, 22, 49]. Specialist providers often expect AYAs with chronic conditions to encounter barriers to finding “adult-focused” providers willing to meet their general healthcare needs [18, 19, 50]. In our study, family physician participants were willing to monitor AYAs during the transition period, but this willingness appeared dependent on being involved prior to transfer, or “*from the beginning”*.

Very few studies have examined the effectiveness of continuous primary care, or having a family physician “*involved from the beginning*”, on transition outcomes [15, 16]. For young children with chronic conditions [51, 52], and older adults [53], regular attendance in primary care has been linked to improved outcomes (e.g., fewer ED visits). Yet, national survey data in the United States suggests less than 50% of AYAs with a chronic condition have a “regular source of care” [54–56]. A recent Canadian population-based study [16] on AYAs with severe mental illness (i.e., schizophrenia, eating disorder, mood disorder) showed two thirds (65.1%) had “continuous” primary care, or seen by the same physician during transition (12–26 years old); those with discontinuous primary care, and no primary care, had increased rates of mental health-related hospitalizations as young adults. Prior to transfer, pediatrician involvement with AYAs with chronic conditions may be a barrier to continuous primary care, as expressed by our participants. Views about the ideal role of pediatricians before and after transition were mixed. In Canada, children with complex needs and those from families with higher income are more likely to have a pediatrician (or subspecialist) as their primary care provider, which creates an added healthcare transition for AYAs who must transfer to a family physician after age 18 [57]. To support the role of family physicians and PHC providers, more evidence is needed on the association of transition health outcomes with continuous primary care, since this may be a protective factor for AYAs during the transition period.

Barriers to engaging AYAs with chronic conditions may exacerbate discontinuous primary care, but are not uncommon to this age group in primary care [18, 54, 58–61]. Primary care providers often report a lack of adolescent training, which may interfere with their ability to engage individuals in this age group [60–62]. Some work suggests AYAs with chronic conditions prefer to access their specialists for certain aspects of their healthcare (e.g., sexual health, mental health) and perceive family physicians as not equipped to manage these issues [17, 22, 63]. Exploring the reasons why AYAs with chronic conditions access, or do not access, primary care is important for informing PHC-based transition interventions and will be a focus of future work.

Coordinated care, between PHC providers and specialists, is critically important at the point of transfer, and also throughout the entire transition continuum (ages 12 to 25) [45]. Barriers to care coordination [64, 65], as described by our participants, emerged as a secondary focus. Further research on solutions for integrating primary and specialty care to improve care coordination, specifically during the transition period [66], is needed. Evidence for the effectiveness of new types of models (e.g., shared care) during transition is lacking and needs to be further explored [21]. Gaining a better understanding of co-management and follow-up practices, by specialists and PHC providers, for AYAs with chronic conditions would be a feasible first step to understanding what strategies exist and could be effectively scaled up.

### Strengths and limitations

We sampled PHC providers from a variety of training backgrounds, in different locations (rural vs. urban), and with varying levels of experience. A limitation of our study was that, in order to answer our research question, we recruited PHC providers with direct experience caring for AYAs with chronic conditions. Many also reported additional training related to AYA health or mental health. The experiences of our participants may not reflect all PHC providers. Indeed, some work has reported high percentages (up to 39%) of primary care providers are reluctant to accept “complex” AYAs, including AYAs with mental health conditions [67–70]. Perspectives from additional PHC providers, for example those with less experience caring for AYAs, are needed to better understand potential barriers of accepting or managing these patients in their practice.

## Conclusions

This study was the first to our knowledge to specifically focus on perspectives of PHC providers about the transition to adult care for AYAs with chronic conditions. Input from family physicians, who follow their patients across the lifespan and provide the majority of primary care, are critical for informing and refining recommended transition practices. Our findings provide insights, from PHC providers themselves, to bolster the rationale for primary care involvement during transitions from pediatric specialty and/or community-based services for AYAs.

## Supplementary information

**Additional file 1: Table S1.** Additional exemplar quotes from participants within each sub-theme or role.

**Additional file 2.**

## Data Availability

The datasets generated and/or analysed during the current study are not publicly available due to data containing possibly identifying, personal information. Data are available for discussion from the corresponding author, on reasonable request.

## Abbreviations

AYA: Adolescents and young adults
PHC: Primary Health Care
PCN: Primary Care Network
