# Supplementary material for: Perspectives from primary health care providers on their roles for supporting adolescents and young adults transitioning from pediatric services
Source: BMC Fam Pract. 2020 Jul 13;21:140. doi: 10.1186/s12875-020-01189-8 (PMC7359255; doi:10.1186/s12875-020-01189-8)
Supplement: Supplementary file 1 — Additional file 1: Table S1. Additional exemplar quotes from participants within each sub-theme or role. [file 12875_2020_1189_MOESM1_ESM.docx]

**Supplemental Materials**

**Table S1:** Additional exemplar quotes from participants within each sub-theme or role

| Theme | Exemplar participant quotes |
| --- | --- |
| Role 1:  Being a “common thread” across the patient’s lifespan (*continuity of care*) | *“I think role of the family doctor is the common thread that is consistent no matter who they’re seeing”* (FP13).  *“It’s all about relationships [and] we have that ongoing relationship with [AYAs]”* (FP3).  *“Maybe they grew up in our practice. We plan to have a long-term relationship with [AYAs], whereas with the specialist, once you’re better you get discharged. But, somebody still has to manage them long-term”* (FP16).  *“I say to the mother, it’s really important that we stay in touch… [AYA’s] not gonna be with pediatric endocrinologist forever… It’s really important because the relationship [AYA] has with me is important. If we just leave it for 5 years, re-connecting at age 16, 17, is a lot harder”* (FP11).  *“This so-called problem [transition] in our world is not a problem….by the time [AYAs] need to transition specialists [at 18], I know their situation well enough [that] it would be unusual they would transition without a plan in place”* (FP11).  *“If they’ve known someone for a long time, and have to be switched just do to age… I think that’s hard for [AYAs] to grasp”* (FP16).  *“The family doctor would see notes back from specialists that make suggestions, and then you see the family and they say, “We didn’t like that plan.” I think that’s less likely to happen with a family doctor who’s going to know that that plan wasn’t going to work to begin with. It’s all about knowing the people over a long period of time”* (FP19).  *“Every 3 months depending on how stable or unstable they are, if they are really stable, then 6 months is reasonable”* (FP3).  *“I am their first point of contact… I may need to refer for more in-depth assessments. But, from the very beginning, it comes through me [for mental health]”* (FP11).  *“If they are stuck about something, I’m an easy call”* (SW18).  *“I think most adolescents lose touch with their family doctor. I think I have a sort of a special interest in them and passion for it? So I stay in touch with them. I’ve delivered lots of the patients I see.”* (FP11)  *“I’m the go-to person. If they’re stuck about something they can call me. They need something they can call me. Easier than phoning the family doctor or the pediatrician. I’m just a constant person for the family if they’re having any struggle.”* (SW18) |
| Role 2:  Providing “holistic care” to AYA *(comprehensive primary care*) | *“Family physicians are generally trained to focus first on the person, and then on the medical problems. So it’s an opportunity for them to be seeing more than the diabetic or the asthmatic, and it’s recognizing that it’s tough enough going through teens, let alone going through it with a chronic illness. I think we’re more attuned to and look for the psychosocial components of children and adolescents*.” (FP5)  *“For everyday things, they rely on us for forms, or some refills or prescriptions or things that fall outside the realm of their specialist care, We’re always there in the background…”* (FP7).  *“We see notes back from specialists that make suggestions. Then we see family and they didn’t do it, ‘cause they didn’t like that plan. A family doctor is going to know that plan wasn’t going to work to begin with. It’s about knowing [AYA] over a long time period”* (FP19).  *“I translate the plan from the specialists to [AYAs] and figure out realistically how to fit [the plan] into their everyday life. You need to consider the social aspects… You can take all the time in the world to pick the perfect medication, but it’s useless if you don’t know if [AYA] is covered, and which pharmacy they’re going to”* (NP15)  *“A lot of times the plan is just ridiculous like its something that’s not accessible to them because its either too expensive or the follow-up is too great and they don’t have the transportation. Its just not realistic. So they can’t follow-up with your beautiful plan that [specialist] crafted and you’re worried about non-compliance”* (NP15).  *“I see a lot of chronic pediatric and adolescent stuff. Especially around anxiety, as well as some depression. But anxiety is the big one. Lot of ADHD as well. And in my mind all these mental health [issues] fall under the umbrella of medical really. There’s no reason for us to have a dichotomy, but anyway but we do.”* (FP11) |
| Role 3:  “We know the families”  *(family-partnered care)* | *“We take care of families”* (FP21).  *“We have advantages, we also know the parents”* (FP13)  *“Complex chronic conditions are best handled in the family doctor’s office, or the office that the rest of the family is seen in”* (FP19).  *“I would know the child is depressed because the mom has terminal breast cancer diagnosis. I see the bigger picture”* (FP21).  *“If one of my colleagues says, “We have a patient who [is] 17 with cystic fibrosis, is on all these enzymes, needs special antibiotics, gets pneumonia… Would you take this patient? And I don’t care for their mother, their father, anybody else in their family… It would be a lot of work… Trying to plug the hole now - why should I?”* (FP11)  *“One of the important things to get care for kids or adults and teenagers is to get the family on board. It’s so important...What I do is to explain again what the specialist has told them. Sometimes they heard it, but they didn’t understand what they said...So I try to ensure that the family engages with the plan, to support this vulnerable child with complex needs so that we can get the best outcome possible*.” (FP21)  *“I know [AYA] is gonna come to me [at age 18] because the parents have stuck with me for 18 years”* (FP7)  *“A lot of motivation to get help has to come from the parents”* (FP15)  *“I understand it could be a risk if you have a child who [is] competent and doesn’t want information disclosed to family especially if they’re making bad choices and they are completely capable of making decisions on their own, but I haven’t come across it”* (FP 21)  *“For those under the age of 18 but needing care, I really do need to establish the mature minor status for them if they’re self referring and not involving their parents... If they are under the age of 18 and they’re not being compliant with disease control, then I need to be pretty clear about what information I have to talk about to their Mum and Dad, because I don’t want to lose the rapport that I have with them [adolescent patient].”* (FP13)  *“Say the mother’s parents aren’t taking good care of the child, and you think it’s neglect, and you’re gonna call social services. “That’s gonna harm your relationship with the parents and therefore you shouldn’t take care of the kid?” That’s bullshit! That could happen in any of your families! That’s part of your legal and ethical responsibility as a [health] professional.”* (FP11) |
| Role 4: “Empowering” AYA patients to develop “personal responsibility” *(developmentally-appropriate care)* | *“These are developing adults. So yes, they’ve got health issues, whether physical or mental, they need to take ownership and be taught to self-manage. But engaging the family is critical”* (FP21).  *“Individualizing”* AYAs’ self-management plans, not a *“cookie-cutter”* approach (SW12)  *“Simple as consent to release information [to parents]”* to assist AYAs with *“adult role”* in care (SW12)  *“If you happen to look after the family as well, and we family doctors usually do, then you know what the parents are like so that really helps to know the optimal involvement of parents”* (FP19).  *“Families concern is [AYA] quality of care and support is going to decline during transition… our role is to try and reassure and try to make it a smooth transition. And advocate for them if we feel it isn’t happening”* (FP21)  *“[AYAs] are allowed to have a confidential relationship with me that doesn’t involve their parent… it allows them to bring up any concerns at all. They don’t have to censor themselves, so I think it promotes full disclosure* (FP5)  *“When they’re 13 plus I start to try to engage them with their healthcare...to, “Okay, you’re going to be an adult. You really have to start to take charge, let’s talk about how we are going to do that.” Depending on how old they are, we’ll have Mum in the room a little bit and then the parent gets kicked out, and then it’s just me and them. “Is there anything you want to tell me that you felt you couldn’t say in front of your Mum?” Within the limits of certain things, I’m not going to report it. Coaching them as an adult and saying, “You need to be taking charge of this, so let’s help you do that.”* (NP15)  *“So I think it’s really important for those kids to acknowledge that it’s tough enough going through this transition, all of a sudden you’re graduating from high school. Do you know what’s lying ahead? Do you know what to do with your life? Gosh! All this other stuff going on can be overwhelming and it’s hard when you’ve got cerebral palsy or whatever else. That’s just a pain in the ass on top of this. Let’s do our best to keep that from dominating your life right now.”* (FP5) |
| Role 5: “Quarterback-ing” for AYA *(coordinating specialist and community services)* | *“Your family doctor is like your quarterback. [We] make sure everybody’s on the field. And if somebody’s gonna go off the field, who’s gonna replace that person if needed. That’s an integral part of our job”* (FP11)  *“Patients know that if they’re gonna fall between the cracks, part of the role of the family doctor is to be there to catch them”* (FP11).  *“[Family medicine] is not just arranging the referrals. I really think the job of family doctors is to do it themselves and get help when needed.”* (FP19)  *“Family medicine is a lot more than [referrals]. It’s not just arranging referrals. I think the job of the family doctor is to do as much as they can themselves and get help where needed”* (FP19).-  *“If they only have one chronic disease, the specialist is sending their consult notes to me and I’m seeing the patient for all their other primary care. If there’s multiple issues then a pediatrician is involved and they are more of the care coordinator until the patient ages up and then I would take on that role”* (FP3)  *“If their specialist has not made very clear follow-up arrangements then it’s left for us…If nobody is going to pick up the ball, then we pick up the ball”* (FP3)  *“It takes a lot of work. I’ll make an effort to go on [EMR] when parents come in and say, ‘Did you know my son had an echo and has seen cardiologists and we’re planning for next cardiac surgery?’ I’ll take some time at end of day [and look up info].”* (FP7)  *“It’s totally up to the family doctor. I think a lot of drs would say well the kid’s not my patient. The kid has a pediatrician… I’m gonna maintain interest with the parents but, I’m not gonna keep up the chart cause youth’s not my patient”* (FP7).  *“No hard and fast rule when I become involved in transition process, so variable”* (FP3).  *“We have lots of services [in rural area], it’s just helping patients navigate where to go”* (SW14)  *“Complex patients can very, very quickly become a big stress to your practice in terms of tremendous amount of extra work that you are doing completely for free in your evening time”* (FP19) |
