# Supplementary material for: Perspectives from primary health care providers on their roles for supporting adolescents and young adults transitioning from pediatric services
Source: BMC Fam Pract. 2020 Jul 13;21:140. doi: 10.1186/s12875-020-01189-8 (PMC7359255; doi:10.1186/s12875-020-01189-8)
Supplement: Supplementary file 2 — Additional file 2. [file 12875_2020_1189_MOESM2_ESM.docx]

Supplementary Materials

Table 1. COREQ 32- Item Checklist

Tong A, Sainsbury P, Craig J. (2007) Consolidated criteria for reporting qualitative research (COREQ): a 32-item checklist for interviews and focus groups. International Journal for Quality in Healthcare: 19:349 – 357

| **Item Number** | **Guide questions/description** | **Reported on page #** |
| --- | --- | --- |
| **Domain 1: Research team and reflexivity** |  |  |
| 1. Interviewer/facilitator | Which author/s conducted the interview? | 7 |
| 2. Credentials | What were the researcher’s credentials? | 1 |
| 3. Occupation | What was their occupation at the time of the study? | 1 |
| 4. Gender | Was the researcher male or female? | Female |
| 5. Experience and training | What experience or training did the researcher have? | 7 |
| 6. Relationship with participants established | Was a relationship established prior to study commencement? | 7 |
| 7. Participant knowledge of the interviewer | What did the participants know about the researcher? | 7 |
| 8. Interviewer characteristics | What characteristics were reported about the interviewer/facilitator? | 8 |
| **Domain 2: Study design** |  |  |
| 9. Methodological orientation and theory | What methodological orientation was stated to underpin the study? | 5-6 |
| 10. Sampling | How were participants selected? | 6 |
| 11. Method of approach | How were participants approached? | 6 |
| 12. Sample size | How many participants were in the study? | 8 |
| 13. Non-participation | How many people refused to participate or dropped out? Reasons? | 8 |
| 14. Setting of data collection | Where was the data collected? | 8,9 |
| 15. Presence of non-participants | Was anyone else present besides the participants and researchers? | 7 |
| 16. Description of sample | What are the important characteristics of the sample? | 8-9, Table 1 (page 22), Table 2 (page 23) |
| 17. Interview guide | Were questions, prompts, guides provided by the authors? | 7 |
| 18. Repeat interviews | Were repeat interviews carried out? |  |
| 19. Audio/visual recording | Did the research use audio or visual recording to collect the data? | 7 |
| 20. Field notes | Were field notes made during and/or after interviews? | 8 |
| 21. Duration | What was the duration of the interviews? | 8 |
| 22. Data saturation | Was data saturation discussed? | 7-8 |
| 23. Transcripts returned | Were transcripts returned to participants for comment and/or correction? |  |
| **Domain 3: Analysis and findings** |  |  |
| 24. Number of data coders | How many data coders coded the data? | 7 |
| 25. Description of the coding tree | Did authors provide a description of the coding tree? | 7 |
| 26. Derivation of themes | Were themes identified in advance or derived from the data? | 5,7 |
| 27. Software | What software, if applicable, was used to manage the data? | 7 |
| 28. Participant checking | Did participants provide feedback on the findings? | 8 |
| 29. Quotations presented | Were participant quotations presented to illustrate the themes/findings? Was each quotation identified? | 9-16, Table S1 (page 27) |
| 30. Data and findings consistent | Was there consistency between the data presented and the findings? | 16-19 |
| 31. Clarity of major themes | Were major themes clearly presented in the findings? | 9,11,12,13,15 |
| 32. Clarity of minor themes | Is there a description of diverse cases or discussion of minor themes? | 11,12,13,15-16, Table 3 (page 24) |
